# Factors associated to the presence of headache in patients with influenza infection and its consequences: a 2010–2020 surveillance-based study

**DOI:** 10.1186/s10194-024-01728-z

**Published:** 2024-02-08

**Authors:** David García-Azorín, Laura Santana-López, José Eugenio Lozano-Alonso, Ana Ordax-Díez, Tomas Vega-Alonso, Diego Macias Saint-Gerons, Yésica González-Osorio, Silvia Rojo-Rello, José M. Eiros, Javier Sánchez-Martínez, Álvaro Sierra-Mencía, Andrea Recio-García, Alejandro Martín-Toribio, Ivan Sanz-Muñoz, Ángel Luis Guerrero-Peral

**Affiliations:** 1 Department of Neurology, Headache Unit, Hospital Clínico Universitario de Valladolid, Hospital Clínico Universitario de Valladolid, Rondilla Santa Teresa Streat S/N, Edificio Rondilla, 47010 Valladolid, Spain; 2 Department of Medicine, Faculty of Medicine, Universidad de Valladolid, Hospital Clínico Universitario de Valladolid, Rondilla Santa Teresa Streat S/N, Edificio Rondilla, 47010 Valladolid, Spain; 3 Fundación Instituto de Estudios de Ciencias de La Salud de Castilla y León, ICSCYL, Valladolid, Soria Spain; 4 Dirección General de Salud Pública, Consejería de Sanidad, Junta de Castilla y León, Valladolid, Spain; 5 Department of Medicine, University of Valencia; INCLIVA Health Research Institute and CIBERSAM, Valencia, Spain; 6 Department of Microbiology, Hospital Clínico Universitario de Valladolid, Valladolid, Spain; 7 National Influenza Centre, Valladolid, Spain

**Keywords:** Headache disorders, Virus diseases, Migraine, Infections, Public Health, Vaccines

## Abstract

Headache is a common symptom of influenza infection; however, its causes and consequences remain uncertain. In this manuscript, we analyzed which demographic and clinical factors were associated with the presence of headache during the course of influenza infection and whether patients with headache had a different prognosis, evaluated by need of hospitalization, sick leave or school absenteeism. The influence study (NCT05704335) was an observational study that analyzed data routinely collected from the Health Sentinel Network between 2010 and 2020. During the study period, 7832 cases were considered, among which, 5275 (67.4%) reported headache. The presence of headache was independently associated with myalgia (2.753; 95%CI: 2.456–3.087, *P* < 0.001), asthenia (OR: 1.958; 95%CI: 1.732–2.214, *P* < 0.001), shivering (OR: 1.925; 95%CI: 1.718–2.156, *P* < 0.001), nasopharyngeal erythema (OR: 1.505; 95%CI: 1.293–1.753, *P* < 0.001), fever (OR: 1.469; 95%CI: 1.159–1.861; *P* = 0.001), sudden onset of symptoms (OR: 1.380; 95%CI: 1.120–1.702, *p* = 0.004), female sex (OR: 1.134; 95%CI: 1.023–1.257, *P* = 0.018), and gastrointestinal symptoms (OR: 1.169; 95%CI: 1.039–1.315; *P* = 0.01). Patients with headache had a sex and age adjusted lower odds of being referred to the hospital (OR: 0.463; 95%CI: 0.264–0.812, *P* = 0.007) and a higher odd of having a sick leave and/or school absenteeism (absenteeism (OR: 1.342; 95%CI: 1.190–1.514, *P* < 0.001). In conclusion, the presence of headache seems associated with symptoms caused by the innate immune response. These findings support a headache pathophysiology linked with the innate immune response. Due to the potential negative consequences and its treatable nature, clinicians should systematically evaluate it and, whenever necessary, treat it too.

**Supplementary Information:**

The online version contains supplementary material available at 10.1186/s10194-024-01728-z.

## Introduction

Influenza viruses are negative-sense, single-stranded RNA viruses of the *Orthomixoviridae* family. They are responsible for acute respiratory infections (ARI) of the upper and lower respiratory tract [1]. Despite the existence of an effective vaccine, influenza accounts for approximately 145,000 deaths per year in the world [2].

Headache is a highly prevalent symptom of influenza, reported by 30–60% of patients [3–5]. It contributes to the disability caused by influenza, causing work and school absenteeism. Prior studies have reported that 91.7% Influenza patients miss at least one workday due to the disease [6], and 39% children miss at least one school day due to the disease [7]. Indirect costs account for about 8 billion United States Dollars (USD) per year, out of the total 11.2 USD annual cost attributed to Influenza [8].

The pathophysiology of headache in acute viral infections is unclear. Some authors argue that it is caused by the innate immune response and the cytokine release [9]. Recent studies point out that some patients with primary headache disorders exhibit higher level of selected cytokines and interleukins [10]. This hypothesis could also be supported if the prevalence of headache would be higher in patients with other systemic symptoms well known to be originated by cytokines, such as fever, myalgia or malaise [4]. In the case of other respiratory viruses, such as the severe acute respiratory syndrome coronavirus 2 (SARS-CoV-2), the presence of headache was associated to the presence of other symptoms, such as myalgia, fever or malaise [11, 12].

The objectives of this study are: 1) to analyze which demographic and clinical factors are associated with the presence of headache during the course of influenza infection; 2) to assess whether patients with headache during the course of influenza infection have a different prognosis than patients without headache, evaluated by need of hospitalization, frequency of medical leave or school absenteeism.

## Methods

### Study design

The *influenCEF* study is an observational analytic study that aims to characterize the frequency, phenotype, risk factors, duration, and pathophysiology of headache as a symptom of influenza infection. In this manuscript, we report the data from the risk-factors part of the study, with case–control study design. The study protocol was registered in ClinicalTrials.gov (NCT05704335). The study was conducted and reported in accordance with the Strengthening in the Reporting in Observational Studies in Epidemiology [13]. East Valladolid Ethics Review Board approved the study (PI 22–2884).

### Study setting

The study setting was Castile and León region, the largest region of Spain, in the northwest area of the country. The study used data from the Health Sentinel Network of Castile and Leon (Red Centinela Sanitaria de Castilla y León, RCSCYL), in collaboration with the Department of Microbiology of the Hospital Clínico Universitario de Valladolid, and the National Influenza Center of Valladolid.

### Study period

A total of ten consecutives surveillance seasons were investigated, from the 2010–2011 season to the 2019–2020 season. An influenza surveillance season is defined as the calendar weeks during which influenza is most likely to circulate at detectable levels (as per sentinel surveillance systems), based on historical knowledge, and usually corresponds to the period between the 40th week of the year until the 20th week of the year after [14]. The reason why the subsequent seasons were not considered was the COVID-19 pandemic, which impacted in the epidemiology and clinical presentation of Influenza, and the change in the study procedures, changing from an in-paper evaluation of patients to a digital registry in electronic health records.

### Study procedures

The study was done using the data routinely collected by the RCSCYL. The Health Sentinel Network of Castile and Leon participates in the Influenza Comprehensive Surveillance Program (*Programa de Vigilancia Integrada de la Gripe*, PVIG) since the 1996–1997 season, which on 2020–2021 became the Acute Respiratory Infections Comprehensive Surveillance Program (*Programa de Vigilancia Integrada de las Infecciones Respiratorias Agudas,* VIGIRA) [15]. It is part of the Acute Respiratory Infections Surveillance System (*Sistema de Vigilancia de Infección Respiratoria Aguda*, SiVIRA) of the Carlos III Health Institute, which is allied to the European Influenza Surveillance Network (EISN) of the European Centre for Disease Prevention and Control (ECDC). This study includes information from the PVIG program, which records patients attended by general practitioners and primary care pediatricians involved in the sentinel network in primary care settings because of an influenza syndrome. All patients who meet the definition criteria of influenza-like illness (ILI) [16] are registered as clinical cases. A nasopharyngeal swab sample is collected from a random sample of all clinical cases, and the diagnosis is confirmed by a multiplex RT-PCR system (Luminex NxTag Respiratory Panel-Luminex; FilmArray Respiratory Panel 2.1. plus; GenexPert Flu/RSV Cepheid), depending on the responsible laboratory. Samples positive to influenza are then sent to the National Influenza Centre for diagnostic confirmation and influenza subtyping, by specific CDC RT-PCR. Active monitoring is done by ca. 100 healthcare providers, including general practitioners, pediatricians, and nurses, covering a population that ranged between 27,461 (season 2015–2016) and 35,081 (season 2017–2018) (supplementary appendix) [17]. Each provider gathers systematically a series of variables in an in-paper questionnaire, at the moment of the patient evaluation. All healthcare providers had been trained in advance in the study variables and data collection, with annual update meetings to refresh all the study procedures.

### Study population

In the present study, patients were included if they fulfilled the ILI criteria [16]: 1) acute respiratory illness with onset during the last seven days; 2) presence of at least one of the following symptoms: fever or low-grade fever, malaise, headache, or myalgia; 3) presence of at least one of the following respiratory symptoms: cough, odynophagia, or dyspnea.

### Study variables

A series of variables are consistently collected by the VIGIRA system, including the season, a series of demographic variables, prior medical history, clinical variables, microbiological variables, and consequences of the disease. No additional variables were obtained. Demographic variables included sex at birth, age at the moment of the infection (stratified into the following age groups: 0–4, 5–14, 15–44, 45–64, 65–74 and 75 or older), and vaccination status at the moment of the infection. Variables related to the prior medical history included prior history of cardiovascular disorders, diabetes mellitus, chronic obstructive pulmonary disease (COPD), prior history of cancer, chronic kidney disease, chronic hepatic disease, or degree three obesity (body mass index > 40). Clinical variables assessed the onset of the disease within 48 h, the sudden onset of the symptoms, the epidemiological contact with another person infected by Influenza, and the presence of fever, shivering, asthenia, myalgia, cough, dyspnea, nasopharyngeal erythema, headache, and gastrointestinal symptoms. Microbiological variables included the oropharyngeal swab test obtention, the result of the test, and the influenza subtype and lineage. Last, the consequences of the disease were assessed in terms of need of hospital referral; the medical leave, in the case of adult patients; work absenteeism, in the case of children, adolescent and youth patients,

### Statistical analysis

Qualitative and ordinal variables are described as frequency and percentage and quantitative variables as mean and standard deviation (SD) or median and inter-quartile range. In the hypothesis testing between cases and controls, two-tailed chi-squared test and Fisher exact test were used in the evaluation of qualitative variables. *P* value was considered statistically significant if < 0.05.

To assess which variables were associated with the presence of headache, first, a direct comparison between the frequency of each variable within cases and controls was done. Second, a logistic regression analysis was done. First, a univariable logistic regression was done, with “headache” as the dependent variable, to evaluate the strength of the association with influenza. Second, all the variables that presented a *P* value < 0.2 were included into a multivariable regression analysis by an “enter” method. Third, the observed *P* values were adjusted for multiple comparisons by False Discovery Rate, according to the Benjamini Hochberg procedure [18].

To evaluate whether headache was associated with a different prognosis, in terms of need of hospital referral, medical leave, or school absenteeism, three regression analyses were conducted, with these variables as dependent variables. Headache was inserted as the only independent variable, and afterwards, all the demographic variables or these that could influence the results, based on the prior literature (age or sex), were included in a multivariable regression, to assess whether headache remained as statistically significant.

In all the regression analyses, odds ratios (OR) and their corresponding 95% confidence intervals (CI) were estimated for all covariates using the backward strategy. Multicollinearity was assessed by the variance inflation factor (VIF) and was deemed as critical when VIF was > 5. Due to the study design and the study procedures, there were no missing data in the database. Compensation for multiple comparisons was done through the procedure of False Discovery Rate with the process of Benjamini-Hochberg [18].

## Results

During the study period, a total of 7832 cases were considered. The number of cases per season varied between 505 (2013–2014 season) and 1146 (2017–2018 season) (Supplementary Table 2).

### Prevalence of headache, and age-adjusted prevalence

**Fig. 1:**
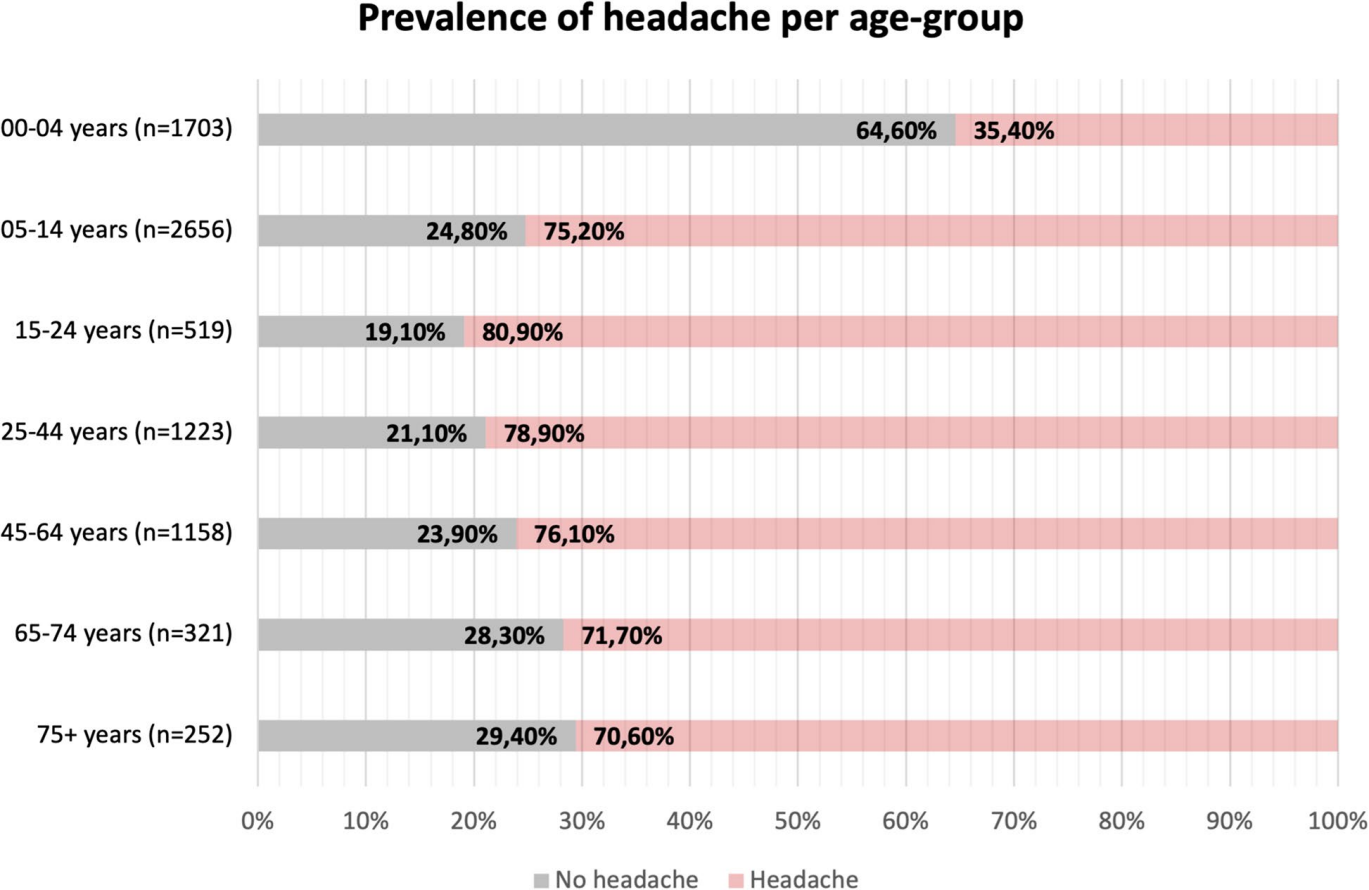
Prevalence of headache per age-group

The prevalence of headache in the entire sample was 5275/7832 (67.4%). Headache had a prevalence > 70% in all age groups except in patients aged 00–04 years, where only 35.4% patients reported it. The highest prevalence was observed in patients aged 15–44 years. Figure 1 shows the prevalence of headache per age-group.

### Differences between patients with and without headache

Patients with headache were more frequently female. Table 1 shows the differences between patients with and without headache. Regarding prior medical history, disorders that were more prevalent within patients with headache included diabetes (+ 1.6%; *P* < 0.001), and COPD (+ 0.5; *P* = 0.034). All the clinical symptoms were more frequent within patients with headache. There were no differences in the microbiological variables. Concerning the consequences of the infection, patients with headache were referred to the hospital less frequently (-0.5%, *P* = 0.006) but required a medical leave more often (+ 4.2%, *P* < 0.001).

**Table 1:** Differences between patients with and without headache

Variable	Entire study sample (*n* = 7832)	Patients without headache (*n* = 2557)	Patients with headache (*n* = 5275)	*P* value
Female sex	3856 (49.2%)	1221 (47.8%)	2635 (50.0%)	0.068
Cardiovascular disorders	193 (2.5%)	64 (2.5%)	129 (2.4%)	0.878
Diabetes	211 (2.7%)	41 (1.6%)	170 (3.2%)	< 0.001
COPD	79 (1.0%)	17 (0.7%)	62 (1.2%)	0.034
Cancer	65 (0.8%)	16 (0.6%)	49 (0.9%)	0.165
Kidney disorders	21 (0.3%)	3 (0.1%)	18 (0.3%)	0.072
Hepatic disorders	14 (0.2%)	4 (0.2%)	10 (0.2%)	0.745
Obesity	67 (0.9%)	15 (0.6%)	52 (1.0%)	0.072
Vaccination	337 (4.3%)	112 (4.4%)	225 (4.3%)	0.814
Onset within 48 h	4573 (58.4%)	1398 (54.7%)	3175 (60.2%)	< 0.001
Epidemiological contact	2964 (37.8%)	884 (34.6%)	2080 (39.4%)	< 0.001
Sudden onset	7325 (93.5%)	2308 (90.3%)	5017 (95.1%)	< 0.001
Fever	7427 (94.8%)	2361 (92.3%)	5066 (96.0%)	< 0.001
Shivering	5129 (65.5%)	1188 (46.5%)	3941 (74.7%)	< 0.001
Asthenia	6009 (76.7%)	1548 (60.5%)	4461 (84.6%)	< 0.001
Myalgia	5248 (67.0%)	1158 (45.3%)	4090 (77.5%)	< 0.001
Cough	6695 (85.5%)	2135 (83.5%)	4560 (86.4%)	0.001
Dyspnea	736 (9.4%)	208 (8.1%)	528 (10.0%)	0.008
Nasopharyngeal erythema	6838 (87.3%)	2128 (83.2%)	4710 (89.3%)	< 0.001
Gastrointestinal symptoms	2123 (27.1%)	628 (24.6%)	1495 (28.3%)	< 0.001
Oropharyngeal swab obtention	2113 (27.0%)	668 (26.1%)	1445 (27.4%)	0.235
Confirmed infection	1194/2113 (56.5%)	376/668 (56.3%)	818/1445 (56.6%)	0.985
A subtype	809/1194 (67.8%)	257/376 (68.4%)	552/818 (67.5%)	0.764
B subtype	385/1194 (32.2%)	119/376 (31.6%)	266/818 (32.5%)	0.823
Hospital referral	49 (0.6%)	25 (1.0%)	24 (0.5%)	0.006
Scholar absenteeism	2205/6624 (33.3%)	765/2315 (33.0%)	1440/4309 (33.4%)	0.759
Medical leave	591 (7.5%)	120 (4.7%)	471 (8.9%)	< 0.001

*COPD* Chronic obstructive pulmonary disease. Differing denominators indicate subgroup analyses

### Factors associated with the presence of headache

In the univariate regression analysis, diabetes, COPD, and all the clinical variables were associated with headache (Table 2). In the multivariable regression, after adjusting for multiple comparisons, the variables that were associated with the presence of headache (Table 2) were female sex (OR: 1.134; 95% CI: 1.023–1.257, *P* = 0.034), sudden onset of the headache (OR: 1.359; 95% CI: 1.100–1.680, *P* = 0.010), the presence of fever (OR: 1.500; 95% CI: 1.180–1.907, *P* = 0.003), shivering (OR: 1.920; 95% CI: 1.713 – 2.152, *P* < 0.001), asthenia (OR: 1.955; 95% CI: 1.728 – 2.212, *P* < 0.001), myalgia (OR: 2.723; 95% CI: 2.427 – 3.055, *P* < 0.001), nasopharyngeal erythema (OR: 1.494; 95% CI: 1.280 – 1.745, *P* < 0.001) and gastrointestinal symptoms (OR: 1.174; 95% CI: 1.043 – 1.321, *P* = 0.018).

**Table 2:** Univariable regression analysis of the variables associated with the presence of headache. Differing denominators indicate subgroup analyses

Variable	Type of analysis	Odds ratio	95% CI lower bound	95% CI upper bound	*P* value	FDR-adjusted *P* value
Female sex	Univariable	1.092	0.994	1.200	0.068	
	Multivariable	1.134	1.023	1.257	0.017	**0.034**
Cardiovascular disorders	Univariable	0.976	0.721	1.323	0.878	
Diabetes	Univariable	2.044	1.448	2.884	< 0.001	
	Multivariable	1.211	0.841	1.743	0.303	0.428
COPD	Univariable	1.777	1.037	3.045	0.036	
	Multivariable	1.209	0.685	2.138	0.514	0.633
Cancer	Univariable	1.489	0.845	2.623	0.168	
	Multivariable	1.050	0.574	1.923	0.873	0.873
Kidney disorders	Univariable	2.915	0.858	9.905	0.086	
	Multivariable	1.922	0.529	6.985	0.321	0.428
Hepatic disorders	Univariable	1.212	0.38	3.869	0.745	
Obesity	Univariable	1.687	0.948	3.003	0.075	
	Multivariable	1.171	0.628	2.185	0.620	0.709
Smoking habit	Univariable	1.163	0.91	1.486	0.228	
Vaccination	Univariable	0.973	0.771	1.226	0.814	
Onset within 48 h	Univariable	1.253	1.139	1.379	< 0.001	
Epidemiological contact	Univariable	1.232	1.117	1.36	< 0.001	
	Multivariable	1.070	0.960	1.193	0.220	0.352
Sudden onset	Univariable	2.098	1.751	2.514	< 0.001	
	Multivariable	1.359	1.100	1.680	0.004	**0.010**
Fever	Univariable	2.012	1.646	2.460	< 0.001	
	Multivariable	1.500	1.180	1.907	0.001	**0.003**
Shivering	Univariable	3.404	3.082	3.76	< 0.001	
	Multivariable	1.920	1.713	2.152	< 0.001	** < 0.001**
Asthenia	Univariable	3.572	3.203	3.983	< 0.001	
	Multivariable	1.955	1.728	2.212	< 0.001	** < 0.001**
Myalgia	Univariable	4.170	3.768	4.614	< 0.001	
	Multivariable	2.723	2.427	3.055	< 0.001	** < 0.001**
Cough	Univariable	1.261	1.106	1.437	0.001	
	Multivariable	0.984	0.846	1.144	0.834	0.873
Dyspnea	Univariable	1.256	1.062	1.486	0.008	
	Multivariable	0.831	0.691	0.999	0.049	0.087
Nasopharyngeal erythema	Univariable	1.681	1.468	1.925	< 0.001	
	Multivariable	1.494	1.280	1.745	< 0.001	** < 0.001**
Gastrointestinal symptoms	Univariable	1.215	1.09	1.354	< 0.001	
	Multivariable	1.174	1.043	1.321	0.008	**0.018**
Sample	Univariable	1.067	0.959	1.187	0.235	
Confirmed infection	Univariable	1.01	0.842	1.212	0.914	
A subtype	Univariable	1.046	0.895	1.223	0.573	
B subtype	Univariable	1.088	0.872	1.358	0.456	

*CI* Confidence interval, *COPD* Chronic obstructive pulmonary disease, *FDR* False discovery rate. Values in bold denotes statistical signification

### Consequences of the presence of headache

In the analysis of the implications of the presence of headache, the presence of headache was associated with a lower odds of hospital referral (OR: 0.463; 95% CI: 0.264–0.812, *P* = 0.007), even after adjusting for sex and age (OR: 0.404; 95% CI: 0.224–731, *P* = 0.003). In the case of medical leave, the presence of headache was associated with a higher odd of medical leave (OR: 1.991; 95% CI: 1.620–2.447, *P* < 0.001) but no higher school absenteeism (OR: 1.017; 95% CI: 0.914–1.132, *P* = 0.759). When adjusted for sex and age, patients with headache had higher odds of medical leave / school absenteeism (OR: 1.342; 95% CI: 1.190–1.514, *P* < 0.001).

## Discussion

In the present study, the factors associated with the presence of headache in patients with influenza were explored. For this, a series of variables were compared between patients with and without headache, including demographic variables, prior medical history of patients, clinical variables and microbiological variables. A subsequent regression analysis was done, to adjust of all the possible confounders. We observed that headache as an influenza symptom was associated with myalgia, asthenia, shivering, nasopharyngeal erythema, fever, sudden onset of symptoms and gastrointestinal symptoms, and with female sex, as the only demographic variable. Second, we explored whether the consequences of the infection were different in patients with headache, and we observed a 60% lower odds of hospital referral and 34% higher odds of medical leave or school absenteeism.

The first striking finding was the high prevalence of headache, which reached two-thirds of the entire study sample. This prevalence seems higher than the observed prevalence in prior studies on Influenza, which observed that 32.4% and 60% out of 37 and 279 patients reported headache during the course of the disease [4, 5]. When compared with SARS-CoV-2, a meta-analysis that included 9573 studies and 28,438 COVID-19 survivors reported a prevalence at onset or hospital admission of 47.1% (95% CI: 35.8 – 58.6%) [19], however, in one study in which neurologists worked at the emergency room and included all the consecutive patients with COVID-19 that were attended by them, the prevalence of headache reached 74.6% of patients [20]. The prevalence was not similar in all age-groups, being higher in patients aged 15–64 years. This has two main implications, related to the pathophysiology and disease consequences. The higher prevalence in younger patients could be related both with the immune-senescence that may exist in old patients [21]. In young children, the verbal description of some symptoms may be challenging, and despite present, headache may not be properly characterized or reported [22]. On the other hand, given the negative consequences of headache, its peak prevalence in people within the most active years, in terms of scholar performance and work, may be particularly relevant [23].

The differences between patients with and without headache should be contextualized. Despite statistically significant, most of these were within the range of 5–10% difference between groups, which may not be that clinically relevant. Given the large sample size, the study had enough power to detect small differences. The exception to this were myalgia, shivering and asthenia, which were 32%, 28% and 24% more frequent within patients with headache, respectively. These symptoms have been associated with the innate immune response and the release of inflammatory mediators [24, 25]. The symptoms that were independently associated with the presence of headache supported the study hypothesis that headache is likely caused by the immune response and the cytokine and interleukin release. Despite this observation results novel in the case of Influenza, prior studies have reported that headache in patients with SARS-CoV-2 is associated with a higher frequency of systemic symptoms [9, 12, 26].

Headache could be associated with a more benign disease course. In the case of SARS-CoV-2, patients with headache had a better prognosis, in terms of mortality or intensive-care admission [9, 11, 27]. Due to this study design, there was no information about patients’ mid-and-long term prognosis, and the only variable that could act as surrogate marker of patients’ outcome was the need of hospital referral. Patients with headache were referred to the hospital 60% less frequently, after adjusting for age and sex, which could support a more benign disease presentation [28].

On the other hand, headache is a disabling symptom, and patients with headache had 34% higher odd of missing work or school. Some aspects, such as the use of symptomatic drugs, including over-the-counter treatments, was not assessed, so we could not evaluate whether these patients had managed the disease adequately [29]. Healthcare providers and even the entire population must be aware of the optimal management of influenza, including drugs that may alleviate the symptoms, the annual vaccination of the vulnerable and exposed population, and the epidemiological measures to minimize the spread of the disease [30, 31]. In our sample, 38% of the cases reported that they suspected the source of their contagion, emphasizing the relevance of isolation measures [32].

The findings of this study should be interpreted in the context of its strengths and limitations. Data was consistently collected by healthcare providers who collaborated with the health sentinel network, carrying out active monitoring. This ensured consistency of data, including the adequacy of study definitions, the representativeness of study participants, and the same method for both cases and controls. The possible effect of patients’ age and sex was anticipated as a possible confounder, and it was controlled to increase the between-group comparability. The main limitation was the selected number of variables that were studied, that did not include some aspects that could be relevant, such as prior history of headache disorders, prior history of headache attributed to acute viral infections. There was no info about the headache phenotype or the presence of other symptoms that are typically associated to headache, such as photo/phonophobia and/or nausea. Patients were evaluated only once, and therefore, there was no information about the patients’ outcome, in terms of mortality, need of intensive-care admission, or need of oxygen therapy. Future studies should consider a longitudinal design to explore this missing piece of the puzzle.

In conclusion, headache is a prevalent symptom of influenza. The presence of headache was independently associated to myalgia, asthenia, shivering, nasopharyngeal erythema, fever, sudden onset of symptoms, male sex, and gastrointestinal symptoms. Patients with headache had a lower odds of hospital referral and a higher odd of medical leave or school absenteeism. These findings support a headache pathophysiology linked with the innate immune response, highlighting the importance of an adequate treatment of it.

### Supplementary Information

**Additional file 1: Supplementary table 1.** Surveyed population per age-group.** Supplementary table 2.** Number of cases per season during the entire study period.
