# Supplementary material for: Factors associated to the presence of headache in patients with influenza infection and its consequences: a 2010–2020 surveillance-based study
Source: J Headache Pain. 2024 Feb 8;25(1):18. doi: 10.1186/s10194-024-01728-z (PMC10854039; doi:10.1186/s10194-024-01728-z)
Supplement: Supplementary file 1 — Additional file 1: Supplementary table 1. Surveyed population per age-group. Supplementary table 2. Number of cases per season during the entire study period. [file 10194_2024_1728_MOESM1_ESM.docx]

**Factors associated to the presence of headache in patients with Influenza infection and its consequences: A 2010-2020 surveillance-based study**

**Supplementary appendix:**

Supplementary table 1: Surveyed population per age-group:

| Season | 00-04 years | 5-14 years | 15-24 years | 25-44 years | 45-64 years | 65-74 years | 75+ years | Total |
| --- | --- | --- | --- | --- | --- | --- | --- | --- |
| 2010-2011 | 2346 | 4537 | 2617 | 8299 | 7290 | 2677 | 4147 | 31914 |
| 2011-2012 | 2314 | 4378 | 2696 | 8403 | 7858 | 2732 | 4402 | 32783 |
| 2012-2013 | 1468 | 3565 | 2521 | 7806 | 7748 | 2576 | 4185 | 29868 |
| 2013-2014 | 1733 | 3986 | 2428 | 7116 | 7549 | 2483 | 3954 | 29250 |
| 2014-2015 | 1874 | 4317 | 2477 | 6742 | 7769 | 2588 | 3705 | 29471 |
| 2015-2016 | 1742 | 4054 | 2319 | 6037 | 7324 | 2487 | 3498 | 27461 |
| 2016-2017 | 1646 | 4113 | 2341 | 6529 | 7501 | 2395 | 3100 | 27624 |
| 2017-2018 | 2901 | 6979 | 2634 | 7196 | 8699 | 2972 | 3701 | 35081 |
| 2018-2019 | 3025,181818 | 7094,454545 | 2656,151515 | 6776,757576 | 8671,969697 | 2890,030303 | 3567,030303 | 34681,57576 |
| 2019-2020 | 3250,333333 | 7136,060606 | 2221,515152 | 5714 | 7391,090909 | 2580,69697 | 3173,272727 | 31466,9697 |

**Supplementary table 2:** Number of cases per season during the entire study period.

| **Season** | **Number of cases** | **Percentage** |
| --- | --- | --- |
| 2010-2011 | 558 | 7.1 |
| 2011-2012 | 650 | 8.3 |
| 2012-2013 | 616 | 7.9 |
| 2013-2014 | 505 | 6.4 |
| 2014-2015 | 905 | 11.6 |
| 2015-2016 | 619 | 7.9 |
| 2016-2017 | 713 | 9.1 |
| 2017-2018 | 1146 | 14.6 |
| 2018-2019 | 1090 | 13.9 |
| 2019-2020 | 1030 | 13.2 |
| Total | 7832 | 100.0 |
